# Alzheimer’s Disease-Associated Neurotoxic Peptide Amyloid-β Impairs Base Excision Repair in Human Neuroblastoma Cells

**DOI:** 10.3390/ijms131114766

**Published:** 2012-11-13

**Authors:** Anne Forestier, Thierry Douki, Sylvie Sauvaigo, Viviana De Rosa, Christine Demeilliers, Walid Rachidi

**Affiliations:** 1 Nucleic Acids Lesions Laboratory, SCIB/INAC, CEA, Joseph Fourier University-Grenoble 1, 17 rue des Martyrs, 38054 Grenoble Cedex 9, France; E-Mails: ann.forestier@gmail.com (A.F.); thierry.douki@cea.fr (T.D.); sylvie.sauvaigo@cea.fr (S.S.); viviana.derosa@ibb.cnr.it (V.R.); 2 INSERM U1055, Joseph Fourier University-Grenoble 1, 38000 Grenoble, France; E-Mail: christine.demeilliers@ujf-grenoble.fr

**Keywords:** neurodegenerative disorders, Alzheimer’s disease, DNA damage, DNA repair, 8oxoGuanine, OGG1, Base Excision Repair, oxidative stress

## Abstract

Alzheimer’s disease (AD) is the leading cause of dementia in developed countries. It is characterized by two major pathological hallmarks, one of which is the extracellular aggregation of the neurotoxic peptide amyloid-β (Aβ), which is known to generate oxidative stress. In this study, we showed that the presence of Aβ in a neuroblastoma cell line led to an increase in both nuclear and mitochondrial DNA damage. Unexpectedly, a concomitant decrease in basal level of base excision repair, a major route for repairing oxidative DNA damage, was observed at the levels of both gene expression and protein activity. Moreover, the addition of copper sulfate or hydrogen peroxide, used to mimic the oxidative stress observed in AD-affected brains, potentiates Aβ-mediated perturbation of DNA damage/repair systems in the “Aβ cell line”. Taken together, these findings indicate that Aβ could act as double-edged sword by both increasing oxidative nuclear/mitochondrial damage and preventing its repair. The synergistic effects of increased ROS production, accumulated DNA damage and impaired DNA repair could participate in, and partly explain, the massive loss of neurons observed in Alzheimer’s disease since both oxidative stress and DNA damage can trigger apoptosis.

## 1. Introduction

Alzheimer’s disease (AD) is an age-dependent neurodegenerative disorder characterized by a progressive decline in behavior and cognition, concomitant with a massive apoptotic loss of neurons from brain regions involved in learning and memory [1]. It is becoming the main cause of dementia in developed countries where, due to a demographic shift toward an elderly population, an increasing number of individuals are affected. The two major pathological hallmarks in AD are neurofibrillary tangles (NFTs) and senile plaques (SPs) [2]. NFTs are intracellular lesions consisting of twisted filaments of the microtubule-associated Tau protein [3], and SPs are extraneuronal aggregates composed of the amyloid-β peptide (Aβ) [4]. Aβ is derived from the pathogenic proteolytic processing of the transmembrane amyloid precursor protein (APP) [5]. Mutations in APP and/or presenilins (PSEN1 and PSEN2) increase this amyloidogenic processing and represent autosomal-dominant familial cases, which account for 5% to 10% of all AD cases [6]. However, most cases of AD are sporadic, and their etiology remains unclear.

Because the brain exhibits a high oxygen consumption rate and lacks strong antioxidant defenses, it is vulnerable to oxidative stress [7]. Therefore, one unifying hypothesis suggests that oxidative processes play contributory or acceleratory roles in many of the salient features of AD (*i.e*., NFTs, SPs and neuronal-selective neurodegeneration). Evidence of increased oxidative stress in AD-affected brains has come from studies showing increased lipid peroxidation [8], increased carbonyl modifications of proteins [9] and increased oxidation of mitochondrial and genomic DNA [10–12]. The cumulative damage over time, especially to DNA, is thought to contribute to selective neuronal cell loss because unrepaired DNA can lead to apoptosis [13]. In addition, recent work has indicated that high levels of DNA damage in neuronal cells can activate the classical cell cycle machinery and S-phase re-entry, which either promote DNA repair or initiate apoptosis [14]. Oxidative damage to DNA by reactive oxygen species (ROS) can lead to DNA-protein or DNA-DNA cross-linking, strand breaks and the generation of oxidized bases, the most common being 8-oxoguanine (8oxoG) [15]. Gabbita *et al*. [16] showed a generalized increase in the levels of purine and pyrimidine base oxidation in the DNA of AD-affected brains, highlighting the fact that 8oxoG is the most frequent of all analyzed modified bases. Another study by Lovell *et al*. [17] revealed an increase in the levels of 8oxoG in cerebrospinal fluid (CSF) from AD patients compared to age-matched controls. Recent publications have shown significant increases in the level of oxidized purines in DNA from peripheral lymphocytes [12] and, more generally, leukocytes [18] from AD patients.

It has been suggested that the accumulation of nucleic acid oxidative lesions in AD could be due not only to an increase in the production of ROS and DNA damage but also to a decreased capacity to repair the modified nucleic acids [19], especially small oxidated base lesions that are typically repaired by the base excision repair (BER) pathway. Briefly, BER is initiated by the removal of the damaged base by substrate-specific DNA *N*-glycosylases, which results in the generation of an abasic site that is subsequently cleaved by an apurinic or apyrimidic (AP) lyase or AP endonuclease. Repair then proceeds either by the short-patch BER pathway, which involves the incorporation of a single nucleotide into the gap by DNA polymerase followed by strand ligation by DNA ligase, or by the long-patch BER pathway, which involves the incorporation of several nucleotides followed by cleavage of the resulting 2- to 8-nucleotide flap and ligation [20,21]. Interestingly, several BER defects have been reported in AD-affected brains. Weissman *et al*. [22] described lower 8oxoG and uracil incisions in the inferior parietal lobule (IPL) of sporadic AD patients and patients with mild cognitive impairment compared to normal age-matched controls, with a corresponding decrease in the expression levels of 8oxoG DNA glycosylase (OGG1) and uracil-DNA glycosylase (UNG). In this study, the authors also report a decrease in the single gap-filling activity in the IPL and a diminished protein expression level of the associated polymerase β (polβ). Similarly, a recent publication showed a decrease in both mitochondrial and nuclear OGG1 activities not only in the IPL but also in the frontal and temporal lobules from AD-affected patients [23]. However, another recent study reported no association between AD-like pathology and DNA repair in transgenic mice carrying the APP751 allele with the so-called double Swedish (K670M/N671L) mutation of APP (APPswe), PSEN1M146V and TauP301L transgenes, or the APPswe transgene alone [24].

To gain further insight into the DNA damage and repair responses related to AD, the present study investigated the effect of Aβ secretion on BER gene expression levels and activity in a neuroblastoma cell line stably transfected with human APP751, which results in an increased secretion of Aβ. Here, we demonstrate that the secretion of Aβ leads to an overall downregulation of *BER*-associated genes. Moreover, the secretion of Aβ inhibits the adaptation of a cell in response to oxidants. BER activities were assessed in cell lysates using two different assays and were drastically decreased in response to increased Aβ secretion.

## 2. Results and Discussion

### 2.1. Results

#### 2.1.1. Cytotoxicity Assay

We used two different oxidants, hydrogen peroxide (H_2_O_2_) and copper sulfate (CuSO_4_), to mimic the microenvironment of degenerative neurons within the AD brain. H_2_O_2_-, and CuSO_4_-induced cytotoxicity was assessed in mock and APP751-expressing cell lines as well as in wild-type untransfected SH-SY5Y, using an MTT assay. At 48 h after plating, cell lines were treated with a range of concentrations of each compound from 100 μM to 1 mM without changing the cell media. The toxicity of both oxidants was not significantly different between the two transfected cell lines (Supplemental Figure S1) except at higher concentrations of copper sulfate (600 μM) where Mock cells were more resistant than APP751, and the rate of cell growth was not affected. Thus, we used the inhibitory concentrations of 10% (IC10) of the mock cell line, which were 115 μM and 350 μM for H_2_O_2_ and CuSO_4_, respectively, as working concentrations for the next studies.

Finally, it is important to mention that there is no effect of the transfection conditions on the vulnerability of SH-SY5Y cells to both oxidants, as the wild-type untransfected SH-SY5Y cells showed the same cell viability profile at the working concentrations (Supplemental Figure S1).

#### 2.1.2. Nuclear DNA Damage Is Increased in APP751-Expressing Cells

**Figure 1:**
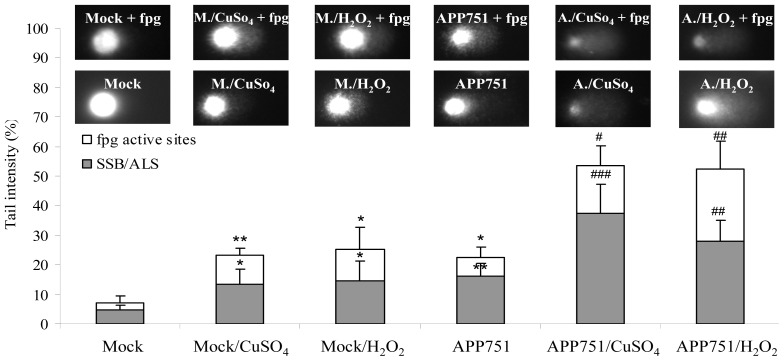
Amyloid-β (Aβ), copper sulfate (CuSO_4_), and hydrogen peroxide (H_2_O_2_)-induced nuclear DNA damage. The comet assay was performed on Mock and APP751-expressing cell lines to detect single-strand breaks (SSB) and alkali-labile sites (ALS), and the formamidopyrimidine DNA glycosylase (fpg) enzyme was further used to get information on the level of oxidized purines (fpg active sites) in each sample. The upper line of the comet pictures represents the fpg-treated slides with a characteristic image for each sample. The second line represents the non fpg-treated slides. Figure 1 displays the graphical representation of the mean tail intensities for each sample, for both fpg active sites (white) and SSB/ALS (grey). Under basal conditions and following exposure to oxidants, the level of DNA damage is higher in the APP751-expressing cells than in the mock ones. * Significantly different (*p* < 0.05) from mock cells, **p < 0.005; ^#^ significantly different (*p* < 0.05) from APP751-expressing cells, ^##^*p* < 0.005, ^###^*p* < 0.0005.

The extent of nuclear DNA damage was characterized using the alkaline comet assay (Figure 1). This assay evaluates the frequency of strand DNA breaks (SSBs) and of alkali-labile sites (ALS). Pretreatement with formamidopyrimidine DNA glycosylase (fpg) allowed us to obtain complementary information on specific DNA lesions, because fpg recognizes 8oxoG and other oxidatively damaged purines. The levels of SSBs and ALS were obtained by quantifying the tail DNA in non-fpg-treated slides and the level of oxidized purines was obtained by subtracting these SSB and ALS tail DNA values from the tail DNA values of the sample corresponding to fpg-treated slides.

Under basal conditions without any exogenous stress, APP751-expressing cells had a higher tail intensity than the mock-transfected cell line (16.08% tail DNA ± 4.43 *versus* 4.75 ± 1.51, *p* = 0.0004). After exposure to CuSO_4_, the tail intensity increased by 8.59% in the mock-transfected cell line (13.34% ± 5.11 *versus* 4.75% ± 1.51, *p* = 0.005) and by 21.3% in the APP751-expressing cell line (37.38% ± 9.86 *versus* 16.08% ± 4.43, *p* = 0.0004). After exposure to H_2_O_2_, the tail intensity increased by 9.71% in the mock-transfected cell line (14.46% ± 6.60 *versus* 4.75 ± 1.51, *p* = 0.01) and by 12.05% in the APP751-expressing cell line (28.13% ± 6.60 *versus* 16.08% ± 4.43, *p* = 0.0008). Thus, the induction of SSBs was more prominent in APP751-expressing cells than in mock cells. Similarly, the level of oxidized purines in the APP751-expressing cell line was higher than in the mock cell line under basal conditions (6.19% ± 3.55 *versus* 2.39% ± 2.35, *p* = 0.03). Exposure to CuSO_4_ increased the fpg-dependent tail intensity in the mock-transfected cell line by 7.69% (10.06% ± 2.27 *versus* 2.39% ± 2.35, *p* = 0.004), and the fpg-dependent tail intensity in the APP751-expressing cell line increased by 9.93% (16.12% ± 6.60 *versus* 6.19% ± 3.55, *p* = 0.03). Exposure to H_2_O_2_ increased the fpg-dependent tail intensity in the mock-transfected cell line by 8.40% (10.79% ± 7.58 *versus* 2.39% ± 2.35, *p* = 0.04), and the fpg-dependent tail intensity in the APP751-expressing cell line was increased by 17.87% (24.06% ± 9.80 *versus* 6.19% ± 3.55, *p* = 0.002).

#### 2.1.3. Mitochondrial DNA Damage Is Increased in APP751-Expressing Cells

**Figure 2:**
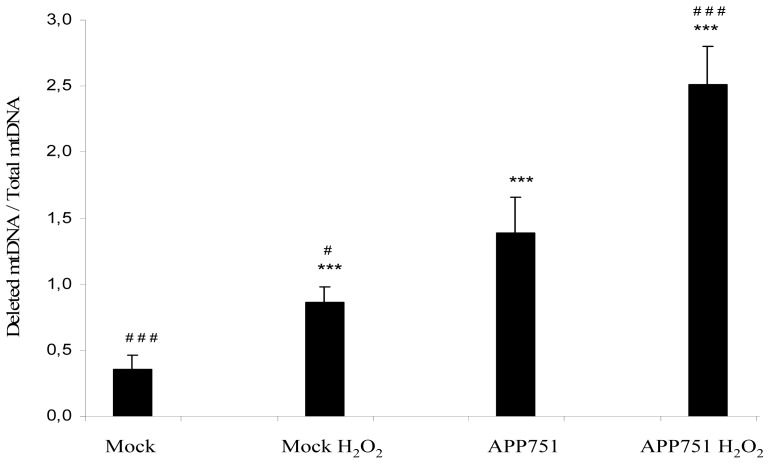
Quantification of a common mitochondrial deletion in mock and APP751-expressing cells after treatment with H_2_O_2_. For both mock and APP751-expressing cells, the ratio of deleted mtDNA to total mtDNA was established using qPCR-based quantification. Under basal conditions and following an H_2_O_2_-induced stress, the APP751-expressing cell line exhibits higher rates of deleted mtDNA than the mock cell line. ***Significantly different (*p* < 0.0005) from mock cells; ^#^ significantly different (*p* < 0.05) from APP751-expressing cells, ^###^*p* < 0.0005.

Mitochondrial DNA damage was characterized by quantifying the common deletion in mitochondrial DNA, a large deletion of 4977 bp, which is the most common and the best characterized mutation in mtDNA. The ratio of deleted mitochondrial DNA versus total mitochondrial DNA was calculated in the mock and APP751-expressing cells, under basal conditions or following treatment with H_2_O_2_ (Figure 2). This ratio was significantly higher in the APP751-expressing cell line than in the mock (1.39 ± 0.27 *versus* 0.36 ± 0.10 *p* = 0.0001) and even higher than in the H_2_O_2_-treated mock (1.39 ± 0.27 *versus* 0.86 ± 0.12 *p* = 0.01). The H_2_O_2_-treated APP751-expressing cells ratio was also significantly higher than in the non-treated APP751-expressing cells (2.51 ± 0.29 *versus* 1.39 ± 0.27 *p* = 0.0003).

#### 2.1.4. Aβ Secretion Leads to an Overall Downregulation of *BER* Genes

**Figure 3:**
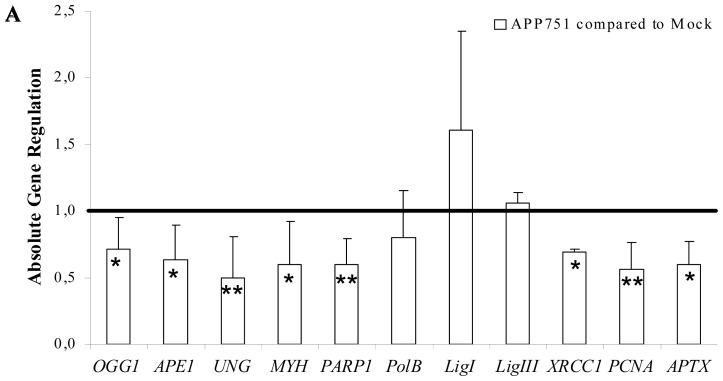

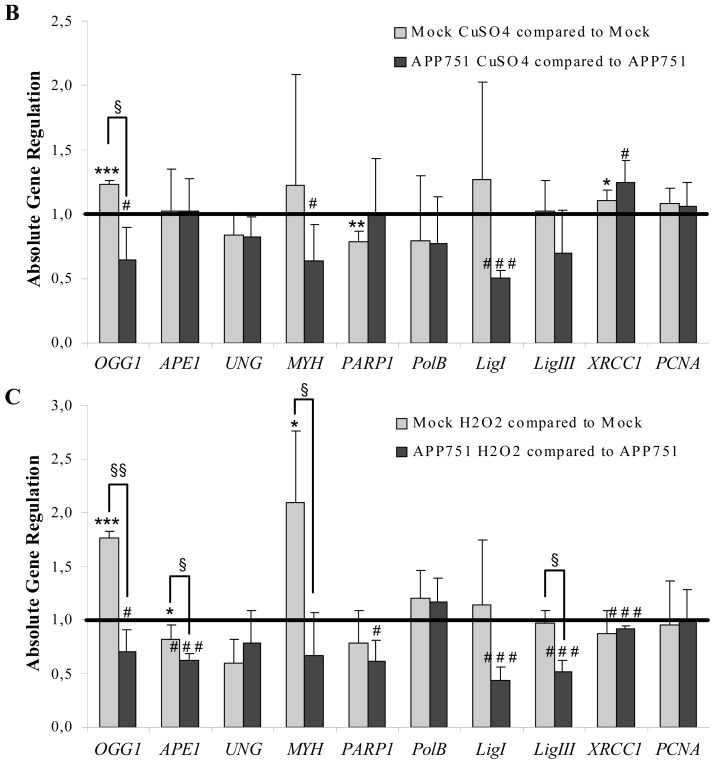
Aβ, CuSO_4_, and H_2_O_2_-induced base excision repair (*BER*) gene expression. Gene expression was investigated using qPCR in mock cells *versus* APP751 cells (**A**), CuSO_4_-treated cells versus untreated cells (**B**) and H_2_O_2_-treated cells versus untreated cells (**C**). Aβ secretion in APP751-expressing cells significantly downregulated 8-oxoguanine DNA glycosylase (*OGG1*), *MutY* homolog (*MYH*), *UDG*, poly (ADP-ribose) polymerase 1 (*PARP1*), X-ray repair cross complementing 1 (*XRCC1*), proliferating cell nuclear antigen (*PCNA*) and aprataxin (*APTX*). The expression profile following CuSO_4_-induced stress was different between mock and APP751-expressing cells. As the most salient features, note that *OGG1* was upregulated in mock cells, while APP751-expressing cells showed downregulation. Mock cells did not overexpress *MYH*, but APP751-expressing cells showed downregulation. The expression profile following H_2_O_2_-induced stress was different between mock and APP751-expressing cells. Mock cells showed upregulation of *OGG1*, while APP751-expressing cells showed downregulation. A few genes were not modulated after H_2_O_2_ exposure in mock cells, but they were downregulated in APP751-expressing cells (*PARP1*, ligase 1 (*LigI*), ligase 3 (*LigIII*), and *XRCC1*). Mock cells overexpressed *MYH*, but it did not appear to be modulated in APP751-expressing cells after H_2_O_2_ exposure. * Significantly different (*p* < 0.05) from mock cells, **p < 0.005, ***p < 0.0005; ^#^ significantly different (*p* < 0.05) from APP751-expressing cells, ^###^*p* < 0.0005 and ^§^ significantly different (*p* < 0.05) from each other, ^§§^*p* < 0.005.

The expression levels of DNA repair enzymes were measured using real-time quantitative PCR. We first investigated the expression level of *BER*-associated genes between the mock-transfected and the APP751-expressing cell lines under basal conditions (Figure 3A). Several *BER*-associated genes were significantly downregulated by the presence of Aβ. For *OGG1*, the human 8oxoG-specific glycosylase, the fold change expression in APP751-expressing cells compared to mock-transfected cells was 0.71 ± 0.23 (*p* = 0.0175). The expression of the *MutY* homolog (*MYH*), another *BER*-associated glycosylase that removes misincorporated adenines in front of 8oxoG during DNA replication, changed 0.59 ± 0.33 fold (*p* = 0.0168) in APP751-expressing cells compared to mock-transfected cells. The expression of *UNG* mRNA in APP751-expressing cells was also reduced compared to mock cells (0.05 ± 0.31, *p* = 0.0047). mRNA levels of apurinic endonuclease 1 (*APE1*) were reduced in APP751-expressing cells (0.63 ± 0.26, *p* = 0.0088). Other *BER*-associated genes, more likely involved in the processing of SSBs than small damaged bases, were also downregulated in APP751-expressing cells compared to mock cells: The expression ratios of poly (ADP-ribose) polymerase 1 (*PARP1*), x-ray repair cross complementing 1 (*XRCC1*) and aprataxin (*APTX*) were 0.59 ± 0.20 (*p* = 0.0016), 0.69 ± 0.02 (*p* = 0.0283) and 0.59 ± 0.18 (*p* = 0.0072), respectively. Genes involved in the final step of long-patch BER, such as proliferating cell nuclear antigen (*PCNA*), were also downregulated in APP751-expressing cells, with an expression ratio of 0.56 ± 0.20 compared to mock (*p* = 0.0012). However, the expression ratios of *polβ*, ligase 1 (*LigI*) and ligase 3 (*LigIII*) mRNA levels were not significantly different between the two cell lines.

We further examined the gene expression profile of the two cell lines following CuSO_4_- or H_2_O_2_-induced stress. After CuSO_4_ treatment (Figure 3B), *OGG1* was significantly overexpressed by the mock cell line (1.23 ± 0.04, *p* = 0.0002), whereas it was downregulated in APP751-expressing cells (0.64 ± 0.25, *p* = 0.0349). Moreover, the expression profile of the two cell lines was also significantly different (*p* = 0.0082). *MYH* mRNA levels were not significantly modified in the mock cell line after CuSO_4_-induced stress but were significantly downregulated in the APP751-expressing cell line (0.64 ± 0.28, *p* = 0.0441). The expression of *PARP1* following CuSO_4_ stress was significantly diminished in the mock cell line (0.79 ± 0.07, *p* = 0.0036), although it was not modified in APP751-expressing cells. *LigI* was not significantly upregulated in mock cells, but it was severely downregulated in APP751-expressing cells (0.50 ± 0.05, *p* = 0.0002). *XRCC1* was overexpressed in both mock (1.10 ± 0.08, *p* = 0.0310) and APP751-expressing (1.25 ± 0.16, *p* = 0.0456) cell lines after CuSO_4_ treatment.

Next, we compared *BER* gene expression between the two cell lines following H_2_O_2_ stress (Figure 3C). *OGG1* mRNA expression levels were significantly upregulated in the mock cell line following stress (1.76 ± 0.07, *p* = 0.00002), while they were downregulated in APP751-expressing cells (0.70 ± 0.21, *p* = 0.0338), and these ratios were significantly different (*p* = 0.0006). *APE1* was slightly downregulated (0.81 ± 0.14, *p* = 0.0398) in the mock cell line and even more diminished (0.62 ± 0.06, *p* = 0.0002) in the APP751-expressing cell line. *APE1* mRNA levels were significantly different in the two cell lines (*p* = 0.0465). *UNG* was significantly downregulated in the mock cell line following hydrogen peroxide treatment (0.60 ± 0.22, *p* = 0.0166) but not in the APP751-expressing cell line. However, the levels of *UNG* in both cell lines after H_2_O_2_ stress were not significantly different. *MYH* was upregulated in the mock cell line after stress (2.09 ± 0.67, *p* = 0.0240), but it was not significantly downregulated in APP751-expressing cells. Nevertheless, following treatment, the two responses were significantly different from each other (*p* = 0.0171). *PARP1* was significantly downregulated in the APP751-expressing cell line after stress (0.61 ± 0.20, *p* = 0.0135). The expression of *LigI*, *LigIII* and *XRCC1* was not modified in the mock cell line after stress but was strongly downregulated in the APP751-expressing cell line, with expression ratios of 0.43 ± 0.13 (*p* = 0.0017), 0.51 ± 0.11 (*p* = 0.0018) and 0.92 ± 0.02 (*p* = 0.0031), respectively. Only *LigIII* expression profiles were significantly different between the two cell lines after stress (*p* = 0.0282).

#### 2.1.5. Aβ Secretion Decreases 8oxoG Excision Capacity

**Figure 4:**
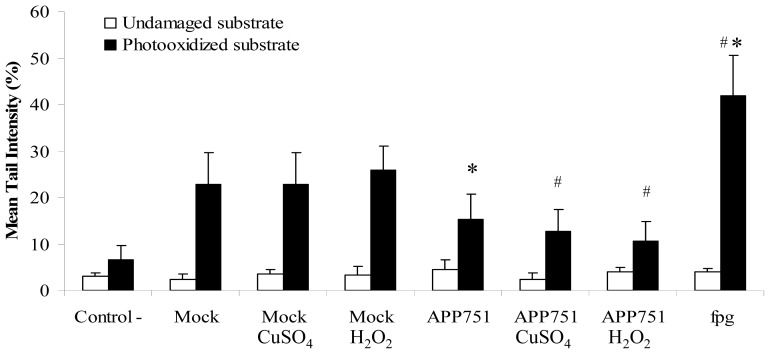
Analysis of 8-oxoguanine (8oxoG) excision activity. The capacity to excise 8oxoG of mock and APP751-expressing cells was analyzed on damaged genomic DNA substrates, using a modified version of the comet assay. There was no increase in the 8oxoG-excision capacity of cell extracts for the mock cell line following either CuSO_4_ or H_2_O_2_-induced stress, but there was a significant decrease in the APP751-expressing cell line following both kinds of stress. * Significantly different (*p* < 0.05) from mock cells; ^#^ significantly different (*p* < 0.05) from APP751-expressing cells.

To investigate whether the capacity to excise major oxidative lesions was also affected, we used a comet-based assay, which assesses the ability of protein extracts to excise 8oxoG from control or specifically damaged cellular substrates. We showed (Figure 4) that protein extracts from the mock cell line had a significantly higher excision potential (22.93 ± 6.72) than the APP751 cell line (15.37 ± 5.36, *p* = 0.0009) under basal conditions. In addition, there was no significant increase in the 8oxoG excision capacity in the mock cell line after copper- or hydrogen peroxide-induced stress. In contrast, we observed that CuSO_4_-induced stress led to a significant decrease (*p* = 0.0151) in excision activity (15.37 ± 5.36 *versus* 12.74 ± 4.58) in APP751-expressing cells, and the difference was even higher after H_2_O_2_ stress (15.37 ± 5.36 *versus* 10.48 ± 4.35, *p* = 0.0060).

#### 2.1.6. Evaluation of Small Damaged Base Excision Capacity Using a Multiplex Assay: ODN Biochip

**Figure 5:**
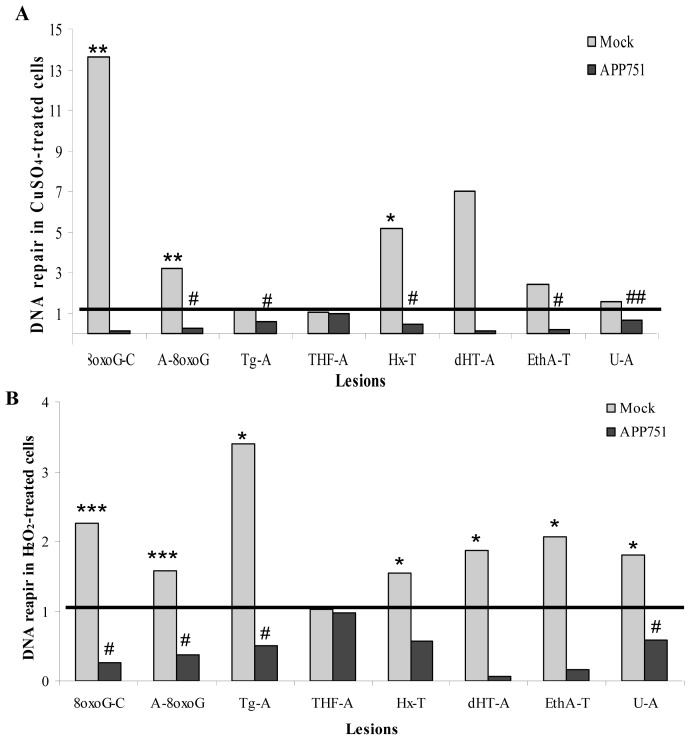
CuSO_4_ and H_2_O_2_-induced excision activity. Nuclear cellular extracts of mock and APP751-expressing cells were tested for their excision capacity on the oligonucleotide (ODN) biochip. The mean percentages of excision capacity (*N* = 3) were generated and the mean ratios of treated/control were calculated for each lesion. Both CuSO_4_ (**A**) and H_2_O_2_ (**B**) treatments stimulated the excision activity in the mock cell line, and they inhibited the excision activity in the APP751-expressing cell line. 8oxoG-C: 8oxoG paired with Cytosine; A-8oxoG: Adenine paired with 8oxoG; dHT-A: dihydrothymidine; EthA-T: ethenoadenine; Hx-T: hypoxanthine paired with Thymine; U-A: uracil paired with Adenine; Tg-A: Thymine glycol paired with Adenine; THF-A: THF paired with Adenine. * Significantly different (*p* < 0.05) from mock cells, **p < 0.005, ***p < 0.0005; ^#^ significantly different (*p* < 0.05) from APP751-expressing cells, ^##^*p* < 0.005.

To obtain further information about the BER capacity in the APP751 cell line, we used a miniaturized assay that allows for the quantification of excision of several DNA lesions, notably specific sugar/nucleobase damage repaired by BER (principles described in Supplemental Figure S2). We investigated the average excision percentage for each lesion in the two cell lines with and without a stress stimulus. Then, for each cell line, we calculated the CuSO_4_- or H_2_O_2_-treated/untreated ratios to focus on the inhibition or induction of the excision capacity of each cell line following stress (Figure 5). The 8oxoG-C excision capacity was enhanced 13.65 and 2.27 fold in the mock cell line following copper and hydrogen peroxide stress, respectively, while it was decreased 0.12 and 0.26 fold in the APP751-expressing cell line following copper and hydrogen peroxide stress, respectively. A-8oxoG recognition was induced 3.19 and 1.58 fold following copper and hydrogen peroxide stress, respectively, in the mock cell line, while it was inhibited 0.28 and 0.38 fold in the APP751-expressing cell line following copper and hydrogen peroxide stress, respectively. The Tg-A excision capacity was increased 1.25 and 3.39 fold in the mock cell line following copper and hydrogen peroxide stress, respectively. In contrast, Tg-A excision capacity was decreased 0.57 and 0.51 fold in the APP751-expressing cell line following copper and hydrogen peroxide stress, respectively. The THF-A excision capacity was not modulated following copper or hydrogen peroxide stress in any cell line: Treated/non-treated ratios were 1.02, 0.99, 1.02 and 0.9 for mock-CuSO_4_/mock, mock-H_2_O_2_/mock, APP751-CuSO_4_/APP751 and APP751-H_2_O_2_/APP751, respectively. The Hx-T excision capacity was enhanced 5.17 and 1.55 fold in the mock cell line following copper and hydrogen peroxide stresses, respectively, while it was decreased 0.45 and 0.57 fold in the APP751-expressing cell line in response to the same treatments. dHT-A repair was increased 7.02 and 1.86 fold following copper and hydrogen peroxide stress, respectively, in the mock cell line, and it was inhibited 0.10 and 0.07 fold in the APP751-expressing cell line following copper and hydrogen peroxide stress, respectively. EthA-T excision capacity was increased 2.39 and 2.07 fold in the mock cell line following copper and hydrogen peroxide stress, respectively. In contrast, EthA-T excision capacity was decreased 0.17 and 0.16 fold in the APP751-expressing cell line following copper and hydrogen peroxide stress, respectively. The U-A excision capacity was enhanced 1.54 and 1.81 fold in the mock cell line following copper and hydrogen peroxide stress, respectively, while it was decreased 0.65 and 0.59 fold in the APP751-expressing cell line following CuSO_4_ and H_2_O_2_ stress, respectively.

### 2.2. Discussion

Alzheimer’s disease is characterized by two major pathologic hallmarks, one of which is the extracellular accumulation of the neurotoxic peptide Aβ [1], which has been reported to generate ROS [25]. In addition to the augmentation of the oxidative stress naturally present in the aging brain [26], it has been proposed that oxidation of various biological macromolecules, including DNA, could play an important role in the loss of neurons in AD [27]. A high level of DNA damage could be particularly deleterious in post-mitotic cells because they do not self-renew through cell proliferation. Therefore the accumulation of oxidative base modifications in nuclear and/or mitochondrial DNA could lead to the selective loss of neurons, and they may also play a significant role in aging and neurodegeneration [26]. It is still not clear how oxidative DNA damage accumulates in tissues of AD patients. Although genetic defects in several DNA repair-related diseases (xeroderma pigmentosum and Cockayne syndrome) are associated with neurodegeneration, it is also not known whether DNA repair and/or the response to DNA damage play significant roles in the pathogenesis of AD [28].

In this study, we explored the consequences of endogenous Aβ secretion coupled to AD-characteristic oxidative stress [29] on the DNA damage and repair responses of an APPSwe-transfected cell line [30]. Indeed, the endogenous secretion of Aβ by the APP751-expressing cells constituted a great advantage for this cell line by avoiding the cytotoxicity associated with the addition of exogenous Aβpeptide and allowed the addition of other compounds that are important to the pathogenesis of AD, such as copper and hydrogen peroxide, at very low doses, thereby more accurately depicting the neuronal environment of the AD-affected brain.

Because an increase in oxidative DNA damage has been widely documented in AD [31,32], we first investigated if the level of DNA damage was also higher in our APP751-expressing cells compared to the mock cell line. To assess the deleterious effects of Aβ on nuclear DNA, we used the well-known alkaline comet assay, which allows for the measurement of SSBs and ALS and the assessment of oxidized purines through the use of the fpg enzyme. We showed that the basal levels of SSBs, ALS and oxidized purines, including 8oxoG, were significantly higher in APP751-expressing cells than in the mock cell line. These findings corroborate the fact that the secretion of endogenous Aβ itself is able to damage DNA through the generation of ROS [33]. Accordingly, we observed that the level of ROS was higher in the APP751-expressing cells (Supplemental Figure S3), *i.e*. Aβ-secreting cells, than in their mock counterparts. Difference in DNA damage between the cell lines was also observed following exposure to a stress. Indeed, treatment of cells by CuSO_4_ or H_2_O_2_ drastically increased the level of DNA damage in the APP751-expressing cell line compared to mock cells. Aβ augmented the effects of copper and H_2_O_2_. It has been shown that copper interacts with APP and Aβ to potentiate neuronal degeneration in AD by promoting deposition and ROS production [34].

Our observation of an increased sensitivity of APP751-expressing cells to oxidative stress was not limited to nuclear but also to mitochondrial DNA, as shown by the frequency of common deletion in mtDNA. Indeed, deleted mtDNA represents a sensitive and early marker for mitochondrial mutations and suffering [35]. We established that the level of deleted mtDNA was considerably higher in the APP751-expressing cells than in the mock cells, and moreover higher than in the H_2_O_2_-treated mock cells, depicting that the Aβ peptide secretion alone was capable of inducing a higher suffering state in the APP751-expressing cell line than an H_2_O_2_ treatment in the mock cell line. Accordingly, the level of deleted mtDNA within the APP751-expressing cells was higher after H_2_O_2_ treatment.

We then hypothesized that SSBs and the oxidized purine-associated DNA repair pathway might also be affected by Aβ. Therefore, we investigated the expression levels of *BER*-associated genes in the two cell lines. When we compared the basal expression profile of *BER* genes between APP751-expressing cells and mock cells, we were surprised to observe that, in spite of increased oxidative damage, the presence of secreted Aβ in the cell culture media induced an overall downregulation of *BER*-associated genes. Interestingly *OGG1* and *MYH*, both of which are involved in 8oxoG repair, were significantly downregulated, although the level of 8oxoG was much higher in APP751-expressing cells than in mock cells according to the comet assay. Interestingly, several changes or defects in the expression of the oxidative stress-sensitive OGG1 have been reported [22,23,36] but not necessarily at the mRNA level. The decrease of *OGG1* in presence of Aβ was also observed in our study at the protein level (Supplemental Figure S4). MYH has barely been studied or associated with AD, perhaps because it is an indirect secondary actor in the repair of 8oxoG, functioning downstream of OGG1 and removing adenine bases misincorporated opposite 8oxoG. Altogether, these results highlight the fact that there is a strong impairment of 8oxoG repair, as *OGG1* is downregulated in the APP751-expressing cell line and the downstream glycosylase *MYH* does not function properly. Another *BER* glycosylase involved in the removal of uracil misincorporated into DNA also appeared to be downregulated in the presence of Aβ. Interestingly, a previous study reported that depletion of UNG in cultured rat hippocampal neurons triggered neuronal apoptosis [37]. *APE1* mRNA levels were also diminished in our experiments. Interestingly, it has been shown that reduced APE1 expression (using siRNA) decreased neuronal cell viability 24 h after exposure to 25–300 μM H_2_O_2_ in culture compared with scrambled siRNA controls [38]. However, previous studies have shown increased abasic site incision activity [39] or no differences between AD samples and age-matched controls [22]. Other BER-associated proteins, particularly those involved in SSB repair, such as PARP1 and XRCC1, were also significantly reduced at the mRNA level in our study, corroborating the findings of Boerrigter *et al*. [40], who showed a decrease in SSB repair in patients with a familial form of AD. Other studies using adult male CD-1 mice have indicated that XRCC1 expression is decreased early following either focal cerebral ischemia or cold injury-induced brain trauma, implying that a failure of DNA repair may contribute to neuronal cell death in these processes [41,42].

The comparison of the expression profile of mock and APP751-expressing cells following stress furthered our understanding. As expected, after CuSO_4_- and H_2_O_2_-induced stress (both generating oxidative stress within the cell), the mock cell line overexpressed genes encoding proteins involved in the *BER* of small damage, including oxidative damage such as *OGG1* and *MYH* (the latter during H_2_O_2_-induced stress only). Interestingly, the expression of APP751 significantly downregulated *OGG1* after both kinds of stress, which is confusing because the level of 8oxoG was higher in CuSO_4_- and H_2_O_2_-treated APP751-expressing cells than in mock cells. These results suggest that, at least at the mRNA level, the presence of Aβ leads to severe deregulation of the *BER* pathway. To extend our observations, we used a comet-based assay to measure the 8oxoG excision activity of our two cell lines. We did not observe a significant increase in excision after stress in the mock cell line, but, basally, the APP751-expressing cell line exhibited a significant reduction in excision capacity, in agreement with the reduced mRNA levels of *OGG1* and *MYH*. In addition, after either copper or hydrogen peroxide-induced stress, the excision capacity of the APP751-expressing cell line was significantly lower than untreated cells. The ODN biochip confirmed these findings, revealing an inhibition of the excision capacity of many small lesions in the APP751 cell line following stress, while mock cells showed an induction of excision capacity after the same stress. Our results suggest that the increase in DNA damage in the APP751-expressing cell line compared to the mock cells could be explained by a decrease in BER-associated enzymes, which is in agreement with a decrease in the excision activity of small lesions typically repaired by BER.

Our data confirm that the Aβ peptide is a source of enhanced oxidative damage to DNA. The impaired BER capacities in AD-affected neurons that we observed worsen this phenomenon by leading to an accumulation of lesions in the genome because of an increased induction and poorer repair of DNA lesions. This dual and synergistic effect of Aβ is likely to facilitate apoptotic cell death because both oxidative stress and unrepaired damage can trigger apoptosis as a result of blocked replication, subsequent replication fork breakdown and DSB generation [43]. The mechanisms underlying the reduced DNA repair capacities in the presence of Aβ remain to be elucidated and may be of crucial interest to avoid this deleterious process.

## 3. Experimental Section

### 3.1. Culture of SKNSH-SY5Y Neuroblastoma Cells (Mock and APP751-Expressing)

We used the human neuroblastoma SKNSH-SY5Y cell line either stably transfected with the empty eukaryotic expression vector pcDNA3 (mock cells which are our control cells) (Invitrogen SARL, Cergy-Pontoise, France) or containing the cDNA of human APP751 (APP751 cell line), which were kindly provided by Dr. Luc Buée [30]. Both cell lines were cultured in Dulbecco’s modified Eagle’s medium/GlutamaxTM supplemented with 10% fetal calf serum (FCS), 1 mM non-essential amino acids, 1% penicillin/streptomycin (Invitrogen) and 400 μg/mL G418 (selection for cells expressing APP751 or the mock vector) in a 5% CO2 humidified incubator at 37 °C.

### 3.2. Cytotoxicity Assay

Mock and APP751-expressing cells (2500 cells per well) were plated in 96-well microtitration plates (Nunc, Dutscher, Brimath, France) in 200 μL of medium and incubated at 37 °C for 48 h. After incubation, two oxidants were tested: hydrogen peroxide (H_2_O_2_, Sigma, Saint-Quentin-Fallavier, France) and copper sulfate (CuSO_4_, Sigma). Cell lines were incubated for 24 h at 37 °C under 5% CO_2_ with 10 concentrations of each tested substance ranging from 100 μM to 1 mM. For treatments, the cell culture media was not replaced because it would remove all of the secreted Aβ from the APP751-expressing cell line. Instead, 20 μL of 11X-concentrated solution was added to the 200 μL already present in each well. At the end of the treatment, the spent cell culture media was replaced with 200 μL of fresh cell culture media, and cells were incubated for another 24 h at 37 °C. At the end of the incubation, cytotoxicity was evaluated using a modified MTT (Sigma) assay [44]. Briefly, 20 μL of MTT (5 mg/mL) in phosphate-buffered saline (PBS, Invitrogen) was added to each well. The medium was removed 2 h later, 200 μL of DMSO (Sigma) was added to dissolve the produced formazan, and the absorbance was measured at 565 nm using a Multiskan RC microplate spectrophotometer (Labsystems, Helsinki, Finland). The absorbance at 565 nm was proportional to the number of viable cells, and survival was calculated as the percentage of specific viability. The concentration of compound leading to 10% cytotoxicity (IC10) of the mock cell line was calculated and used as a working concentration for both mock and APP751-expressing cell lines.

### 3.3. Preparation of Frozen Pellets

Mock and APP751-transfected cell lines were plated in 75-cm^2^ flasks (Becton Dickinson Biosciences, Pont-de-Claix, France) and incubated for 48 h before treatment. Each cell line was then treated with the amount of either H_2_O_2_ or CuSO_4_ corresponding to the IC10 of the mock cell line by adding 1.5 mL of the 11X-concentrated solutions to the 15 mL of culture media already present in the flask. In control flasks, 1.5 mL of water alone was added. Cell lines were incubated for an additional 24 h and then collected by trypsinization, recovered, counted and pelleted by centrifugation at 300*g* for 10 min. The required amount of cells for each appropriate experiment was resuspended in FCS supplemented with 10% DMSO, gently frozen to −80 °C and stored in liquid nitrogen or dry-frozen.

### 3.4. Quantification of the Common Deletion in Mitochondrial DNA

The procedures described in the Sections 3.4.1, 3.4.2 and 3.4.3 were previously published [35].

#### 3.4.1. Lysate Preparation

Dry-frozen pellets of Mock and APP751-expressing cell lines (1 × 106 cells each), treated or not with H_2_O_2_, were disrupted using a Retsch MM 301 mixer mill (2 min, 30 Hz, 2 mm tungsten carbide bead) in 1 mL of 1× lysis buffer (Tween 20 0.005 *v*/*v*; NP40 0.05% *v*/*v*; Tris HCl 10 mM pH 8.3) and proteinase K was added to a final concentration of 0.1 mg/mL. The samples were incubated at 56 °C for 30 min and the proteinase K inactivated by heating at 98 °C for 15 min.

#### 3.4.2. qPCR Amplification of Nuclear, Total Mitochondrial and Deleted Mitochondrial DNA

Twenty microLiter of the lysate was diluted into 100 μL of 0.5× lysis buffer and sonicated for 10 min. Again, 14 μL of the previously diluted and sonicated lysate was diluted into 8 μL of freshly sonicated 1× lysis buffer and 48 μL H_2_O (final concentration: 0.214× lysis buffer). These dilution steps were made in order to homogenize the detergent present in the lysis buffer and to obtain reproducible qPCR efficiency. The LightCycler FastStart DNA Master SYBR Green I kit (Roche) was used to perform qPCR analysis. Each qPCR reaction was carried out using 5 μL of final lysate, 7 mM MgCl_2_ and 0.4 μM for both Forward and Reverse primers (for primers sequences of GAPDH, total mitochondrial DNA (Tot. mt) and deleted mitochondrial DNA (Del. mt) see [35]). The qPCR experiments were performed on the LightCycler (Roche) and composed of a first segment of 1 cycle of 600 s at 95 °C to activate the Taq polymerase, a second segment of 45 cycles of three successive steps of 20 s at 95 °C, 5 s at 54 °C and 8 s at 72 °C and of a third and last segment of one cycle of three successive steps of 20 s at 95 °C, 30 s at 68 °C and 30 s at 95 °C to acquire the fusion curve. GAPDH, Tot. mt and Del. mt were amplified in triplicate in the following samples: the mock and APP751-expressing cells treated or not with H_2_O_2_, and a mix of these four conditions used as a calibrator (cal). This experiment was conducted twice on two distinct biological replicates. The comparative threshold cycle method was used to perform the relative quantification. Thus, Tot. mt and Del. mt abundance were calculated relative of GAPDH for each sample respectively through the ratio *A*1 = (*E*_Tot. mt_)^ΔCt Tot.mt (cal−sample)^/ (*E*_GAPDH_)^ΔCt GAPDH (cal−sample)^ and the ratio *A*2 = (*E*_del. mt_)^ΔCt del. mt (cal−sample)^/(*E*_GAPDH_)^ΔCt GAPDH (cal−sample)^. While *E* corresponding to the corrected PCR efficiency obtained through LinRegPCR software. Then, we obtain the quantification of Del. mt relative to Tot. mt by the ratio *A*2/*A*1.

### 3.5. Alkaline Comet Assay

The alkaline single-cell gel electrophoresis assay was used to determine the presence of single-strand breaks (SSBs) and alkali-labile sites (ALS). Additional information on the level of oxidized bases was gathered from the quantification of formamidopyrimidine DNA glycosylase (FPG)-sensitive sites using a modified version of the comet assay. The assay was essentially conducted as described by Sauvaigo *et al*. [45]. Briefly, frozen pellets of mock and APP751-expressing cells treated or not by CuSO_4_ and H_2_O_2_ were embedded in low-melt agarose (Sigma) at 37 °C (final concentration 0.6% in PBS) and spread on microscope slides coated with one dried layer of 1% normal agarose (Sigma) in PBS. After gelling on ice, the slides were immersed in a lysis solution (2.5 M NaCl, 0.1 M EDTA-Na2, 10 mM Tris, 1% sodium sarcosinate, 1% Triton X-100, and 10% DMSO, pH 10; Sigma) at 4 °C overnight in the dark. The slides were neutralized with three washes (5 min) in 0.4 M Tris–HCl (Sigma), pH 8, and equilibrated in the FPG digestion buffer (3 × 5 min in 0.1 M KCl, 0.5 mM EDTA-Na_2_, and 0.04 M Tris-HCl, pH 8). Digestion with FPG (1.7 μg/mL, 100 μL per slide) for the detection of oxidized purines was performed for 45 min at 37 °C. Control slides were treated with the FPG digestion buffer alone. After digestion, the slides were transferred to an electrophoresis tank filled with electrophoresis buffer (0.3 M NaOH and 1mM EDTA-Na_2_) for 30 min at room temperature. Electrophoresis was performed for 30 min at 25 V and 300 mA. After migration, the slides were rinsed in neutralization buffer (3 × 5 min, room temperature). Finally, slides were stained with ethidium bromide (10 μg/mL), and comet analysis was performed using the image analysis Comet IV software (Perceptive instrument, Haverhill, UK). For each sample, the average tail intensity was determined from the analysis of 50 comets of each slide of a triplicate experiment.

### 3.6. Reverse Transcription (RT) and Real-Time Quantitative PCR (qPCR) Analysis

Total RNA was extracted from each sample using the GenElute mammalian total RNA miniprep kit (Sigma) following the manufacturer’s protocol with the optional DNase treatment step. RNA quality was assessed using native gel electrophoresis. Total RNA was considered intact if two sharp *28S* and *18S* rRNA bands were visualized. RNA (2 μg) from each condition was reverse-transcribed to cDNA (SuperscriptTM II Reverse Transcriptase, Invitrogen), and 20 ng of each cDNA template was used in PCR reactions with gene-specific primers. qPCR was performed in an MX3005p Multiplex Quantitative PCR system (Stratagene, La Jolla, CA, USA) using MESA Blue qPCRTM Mastermix Plus for SYBR^®^ Assay Low ROX (Eurogentec SARL, Angers, France). The integrity of amplification, indicated by a single melt peak for each product, was verified using a dissociation curve analysis. As an endogenous control in RT-PCR analysis, we tested three different housekeeping/reference genes for optimal normalization of the target genes. 18S Ribosomal 1 (*S18*), glyceraldehyde-3-phosphate dehydrogenase (*GAPDH*) and cyclophilin B (*CycloB*) were amplified in triplicate in each sample and condition during the same qPCR run. Corresponding cycle threshold (Ct) values were exported to BestKeeper [46], an Excel-based pair-wise correlation tool that analyzes variability in the expression of individual genes (standard deviation (*SD*) and coefficient of variation) and generates a weighted expression index (the BestKeeper Index) in the form of a geometric mean of Ct values from several candidate reference genes. Then, the correlation between each candidate and the index was calculated, describing the relationship between the index and the contributing candidate reference gene by the Pearson correlation coefficient (*r*), coefficient of determination (*r^2^*) and the *p* value. Individually, *S18*, *GAPDH* and *CycloB* showed a SD less than one. The analysis of the Pearson coefficient correlation showed a strong correlation for all candidates. Individual *SD* and Pearson coefficient correlations of the three genes were tested in each PCR run. Target gene mRNA expression was normalized to the expressed housekeeping genes *S18*, *GAPDH* and *CycloB* using the Relative Expression Software Tool 2006 [46], which uses the pair-wise fixed reallocation randomization test as a statistical model. Corresponding p values were analyzed to evaluate the significance of each expression ratio after each PCR run, but we used Student’s *t*-test for comparing the means of expression ratios between two conditions after three to seven qPCR runs, which is equivalent to three to seven biological replicates, for each tested target gene.

### 3.7. Comet-Based DNA Repair Assays

#### 3.7.1. Substrate Preparation

LnCap cells were grown in RPMI-1640 supplemented with 10% FCS and 1% penicillin/streptomycin (Invitrogen) in a 5% CO_2_ humidified incubator at 37 °C. Sub-confluent cultures of cells were incubated with 1 μM riboflavin (Sigma) at 37 °C for 20 min and then UVA irradiated at 10 J/cm^2^ on ice to induce 8oxoG, a substrate for BER. Cells were then collected by trypsinization, suspended in freezing medium (RPMI-1640 with 20% FCS and 10% DMSO) at 5 × 105 cells/mL, frozen slowly to −80 °C and stored in liquid nitrogen.

#### 3.7.2. Preparation of Whole-Cell Extracts

Liquid nitrogen-stored frozen aliquots of mock and APP751-expressing cells were centrifuged at 300*g* at 4 °C for 5 min. The supernatant was then removed, leaving a dry pellet that was subsequently frozen by immersion in liquid nitrogen and resuspended in 33 μL of extraction buffer (45 mM HEPES, 0.4 M KCl, 1 mM EDTA, 10% glycerol, 0.1 mM DTT, and 0.25× Triton, pH 7.8). The mixture was then vortexed for 30 s, incubated for 5 min on ice and centrifuged at 14,000*g* for 5 min at 4 °C. The supernatant was then combined with a 1:5 volume of reaction buffer (40 mM HEPES, 0.1 M KCl, 0.5 mM EDTA and 0.2 mg/mL BSA, pH 8).

#### 3.7.3. Measurement of DNA Repair Capacities

The principle of the assay is that the repair enzymes present in the cell extracts recognize the damaged DNA of the gel-embedded nucleotide substrates (damaged cells) containing high levels of 8oxoG. Repair processes incise the DNA substrate and cause single-strand breaks that can be determined using single-cell alkaline gel electrophoresis. Therefore, an increase in the tail is proportional to the DNA repair capacities of 8oxoG lesions by the protein extracts.

Frozen pellets of the photooxidized LnCap substrate were embedded in 0.6% low-melting agarose, spread on microscope slides previously precoated with 1% normal melting point agarose, gelled on ice and immersed in lysis solution overnight at 4 °C. The slides were neutralized and equilibrated in the reaction buffer (3 5-min incubations in 40 mM HEPES, 0.1 M KCl, 0.5 mM EDTA, and 2 mg/mL BSA, pH 8). A total of 50 μL of extract was placed on each slide, covered with a cover glass and incubated for 30 min at 37 °C. As a positive control, we used the repair enzyme FPG (1.7 μg/mL, 100 μL per slide), and we used 50 μL of a mix of extraction and reaction buffers as a negative control. After digestion, the slides were transferred to an electrophoresis tank filled with electrophoresis buffer prechilled at 4 °C. The slides were left at room temperature for 30 min, and electrophoresis was subsequently performed for 30 min at 25 V and 300 mA. After migration, the slides were rinsed in neutralization buffer (3 5-min washes, room temp.) and stained with ethidium bromide.

### 3.8. Oligonucleotide (ODN) Biochip

#### 3.8.1. Preparation of Nuclear Extracts

We prepared nuclear extracts as described previously [47]. Briefly, thawed cells were washed twice in ice-cold PBS. The pellets were suspended in 1 mL of ice-cold buffer A (10 mM HEPES, pH 7.9, 1.5 mM MgCl_2_, 10 mM KCl, 0.01% Triton X-100, 0.5 mM DTT, and 0.5 mM PMSF). After 20 min on ice, lysis was completed by vortexing the tube for 30 s. Complete lysis was confirmed using trypan blue exclusion. Nuclei were recovered by centrifugation for 5 min at 5000 rpm at 4 °C and suspended in 25 μL of ice-cold buffer B (10 mM HEPES, pH 7.9, 1.5 mM MgCl_2_, 400 mM KCl, 0.2 mM EDTA, 25% glycerol, 0.5 mM DTT, protease inhibitors (Complete-mini, Roche, Meylan, France) and 0.5 mM PMSF). Lysis of the nuclear membranes proceeded for 20 min on ice. Two cycles of freeze-thaw in liquid nitrogen for 30 s and incubation at 4 °C for 5 min were performed. The extracts were centrifuged for 10 min at 13,000 rpm at 4 °C, and the supernatant was recovered. Aliquots of 10 μL were stored frozen at −80 °C. The BCA kit (Interchim, Montluçon, France) was used to measure protein concentration, which was typically 1 mg/mL.

#### 3.8.2. Preparation of Lesion ODN Biochip

The multiplexed ODN array, which had previously been described [48], was used. A schematic representation of its principles and use is given in supplementary data (Supplemental Figure S2). Rapidly, biotinylated support ODNs (at optimized concentrations: 1–1.5 μM in PBS) were printed on streptavidin glass slides (Xantec bioanalytics GmbH, Germany) in duplicate in a 24-well format. The wells were subsequently individualized by setting the slides into ArrayIt^®^ microplate hardware. Duplexes pre-formed through the specific hybridization of one Cy3-labeled lesion ODN and one long ODN were hybridized on support ODNs for 1 h at 37 °C in a total volume of 80 μL. Each long ODN has a part complementary to a lesion ODN and a part complementary to a specific support ODN. This latter part directs the hybridization onto a specific location through the support ODN. Slides were then rinsed three times for 5 min with 80 μL of excision buffer (10 mM HEPES/KOH, pH 7.8, 80 mM KCl, 1 mM EGTA, 0.1 mM ZnCl_2_, 1 mM DTT, and 0.5 mg/mL BSA).

Each well contained a control ODN and eight lesion-containing ODNs in duplicate: 8oxoG paired with cytosine (8oxoG-C), adenine paired with 8oxoG (A-8oxoG), thymine glycol (Tg) paired with adenine (Tg-A), tetrahydrofuran as an AP site substrate equivalent paired with adenine (THF-A), hypoxanthine paired with thymine (Hx-T), dihydrothymidine paired with adenine (dHT-A), ethenoadenine (EthA-T) and uracil paired with adenine (U-A).

As much as possible, the lesions were in the same sequence context to limit the possible influence of surrounding bases on cleavage efficiency.

#### 3.8.3. Excision Reaction

On each 24-well slide we set six control wells containing excision buffer alone and 18 reaction wells for the excision reactions with the extracts. Nuclear extracts (20 μg/mL in 80 μL of excision buffer) were added to the wells in duplicate and incubated at 30 °C for 30 min. The excision reaction was stopped by washing the slides three times for 5 min in PBS/0.2 M NaCl/0.1% Tween 20. The residual fluorescence of each spot was quantified at 532 nm using a Genepix 4200A scanner (Axon Instrument, Molecular Devices, Sunnyvale, CA, USA) and the Genepix Pro 5.1 software (Axon Instrument). Results were normalized as described previously [47]. For each slide, we used the normalized fluorescence level of the control wells as a reference (repair buffer alone), and we set the fluorescence level of each lesion ODN of the control well to 100. The excision rate of each lesion was then calculated as a percentage of the fluorescence of the corresponding lesion ODN in the control wells. In addition, each well contained a control ODN (without any lesion) that was used to assess the presence of any nonspecific degradation activity in the extracts. The maximum level of degradation was about 10% for the control ODN. For the calculation of the final lesion ODN cleavage percentage, we then applied a correcting factor that took into account the possible control ODN degradation. Consequently, the final lesion ODN excision percentage was 100 × (1 − percentage of fluorescence of lesion ODN/percentage of fluorescence of control ODN).

The results are presented as the ratio of cleavage in treated *vs*. untreated cells for each lesion.

### 3.9. Statistical Analysis

All of the statistical studies were performed using the Student’s *t*-test. Differences were considered significant when the *p* value was <0.05. The number of replicates in each experiment and the number of independent experiments are indicated in the respective figures.

## 4. Conclusions

In this study we showed that the endogenous secretion of Aβ in a human APP751-expressing neuroblastoma cell line led to an increase in oxidative DNA damage and concomitantly to an overall downregulation of *BER* genes compared to the mock control. We also observed that Aβ prevented the APP751-expressing cells from adapting and responding to additional oxidants. These results were obtained at the levels of both gene expression and protein activities. However, we are aware that in the future we need to validate our results on another genetically modified cell line or terminally differentiated neurons to validate this based cell-study. The BER impairment we report in our APP-751 cell line is concordant with numerous other studies describing a BER decrease in AD-affected brains. BER defects could lead to an accumulation of DNA damage over time and further lead to the massive apoptotic neuronal death responsible for Alzheimer’s disease.
